# Optimizing labor duration with pilates: evidence from a systematic review and meta-analysis of randomized controlled trials

**DOI:** 10.1186/s12884-024-06785-5

**Published:** 2024-08-31

**Authors:** Arezoo Haseli, Farideh Eghdampour, Hosna Zarei, Zahra Karimian, Dara Rasoal

**Affiliations:** 1 Family Health and Population Growth Research Center, Health Institute, Kermanshah University of Medical Sciences, Kermanshah, Iran; 2 Department of Midwifery, Islamic Azad University, Marand, Iran; 3 Student Research Committee, Kermanshah University of Medical Sciences, Kermanshah, Iran; 4 Department of Midwifery, Nursing and Midwifery Faculty, Kashan University of Medical Sciences, Kashan, Iran; 5 School of Health and Welfare, Dalarna University, Högskolegatan 2, Falun, 79188 Sweden

**Keywords:** Exercise, Physical activity, Pilates, Pregnancy, Review, Meta-analysis

## Abstract

**Background:**

Pilates has captured interest due to its possible advantages during pregnancy and childbirth. Although research indicates that Pilates may reduce labor duration, alleviate pain, and improve satisfaction with the childbirth experience, consensus on these outcomes remains elusive, underscoring the necessity for additional studies.

**Aim:**

This systematic review and meta-analysis aimed to assess the impact of Pilates exercises on labor duration among pregnant women.

**Methods:**

The online database was searched to yield the literature using the terms of ‘Pilates’, ‘childbirth’, and ‘labor duration’, and similar terms including PubMed, Clinical Key, Scopus, Web of Science, Embase, and Cochrane Database of Systematic Reviews up to June 25, 2023. Studies were considered eligible if they were randomized or clinical controlled trials (RCTs/CCTs) published in English, focusing on healthy pregnant women without exercise contraindications. The studies needed to include interventions involving Pilates or exercise movement techniques, a comparison group with no exercise, and outcomes related to labor duration, the period of the active phase, and the second stage of delivery.

**Results:**

Eleven studies, totalling 1239 participants, were included in the analysis. These studies provided high-quality evidence from exercise only RCTs/CCTs. The findings indicated a significant reduction in the active phase of labor (8 RCTs, *n* = 1195; Mean Difference [MD] = -56.35, 95% Confidence Interval [CI] [-89.46 to -23.25]) and overall labor duration (8 RCTs, *n* = 898; MD = -93.93, 95% CI [-138.34 to -49.51]) in pregnant women who engaged in Pilates exercises compared to those who did not but doesn’t affect on the duration of the second stage of labor (7 RCTs, *n* = 1135; MD = -0.11, 95% CI [-7.21 to 6.99]).

**Conclusions:**

While this review primarily addresses the effects of Pilates on healthy and low-risk pregnant women, the findings suggest a potential role for Pilates in shortening labor duration. Therefore, engaging in Pilates or similar physical activities is recommended for pregnant women to potentially facilitate a more efficient labor process.

**Supplementary Information:**

The online version contains supplementary material available at 10.1186/s12884-024-06785-5.

## Introduction

Pilates is characterized as a distinct form of resistance and strength training [1], and has been described as a “healing exercise” [2]. The primary objective of Pilates is to establish muscular strength and flexibility by fortifying weaker muscles and augmenting the elasticity, and preparing the body of a pregnant woman for childbirth and postnatal recovery [3–5]. Pilates, a form of exercise increasingly popular in health promotion programs worldwide, especially during pregnancy, emerges as a promising activity in this context [6]. Several studies have investigated the impact of Pilates on various aspects of pregnancy and childbirth [3, 7]. Some studies suggest that Pilates may reduce perceived stress during pregnancy, potentially creating mental space for bonding with the unborn child and enhance maternal satisfaction [7–9]. This exercise method is recommended as a particularly relevant physical activity for healthy pregnant women, owing to its capacity to enhance postural coordination, flexibility, balance, and overall quality of life [8, 10]. Moreover, the Pilates method has been used by pregnant women to improve the physical and psychological outcomes of pregnancy, potentially reducing pain such as back pain and pain during labor [11, 12]. Additionally, Pilates has been reported to have no negative effects on gestational age, birth weight of infants, and APGAR scores [13]. However, the comparison of the effects of clinical Pilates exercises with and without childbirth training on pregnancy and birth results did not show any negative impact on the APGAR score [14].

Contemporary research posits that sedentary women should commence physical activity during pregnancy to mitigate these risks [15]. At the same time, there has been an increasing focus in recent years on enhancing the childbirth experience and employing techniques to reduce labor duration [16]. Another aspect that is described from previous studies is the established correlation between protracted labor and heightened risks of mortality, as well as maternal and perinatal complications [17–20]. These complications encompass a range of issues, including increased maternal fatigue, the necessity for induction and cesarean section, instrumental delivery, uterine atony, maternal mortality, elevated fetal distress, hypoxia, low apgar scores, and ultimately, fetal demise [21].

However, the effects of Pilates on childbirth outcomes are still a topic of debate. Some studies have shown that Pilates exercise alone had no significant effect on increasing the rate of natural childbirth in primiparous women [9]. Similarly, there is no consensus on the effects of Pilates methods with voluntary pelvic floor muscle contractions in pregnant women [22].

Research has also demonstrated that Pilates exercises applied during pregnancy could improve women’s core stability and balance levels and reduce their fear of childbirth [23]. While some studies suggest the potential benefits of Pilates on labor duration, there is still a lack of consensus on its overall impact. This systematic review and meta-analysis have been conducted with the objective of elucidating the impact of Pilates exercise on the duration of labor.

## Methods

This systematic review has been meticulously conducted in alignment with the established protocols and recommendations outlined in the Preferred Reporting Items for Systematic Reviews and Meta-Analyses (PRISMA) guidelines [24].

### Literature search and data collection

This research was conducted through June 2023 using the following search terms: (childbirth OR pregnancy OR pregnant OR labor OR obstetric) AND (Pilates OR physical activity OR exercise OR movement techniques) AND (duration of labor OR length of labor OR length of delivery). The research was carried out using the following databases: PubMed, Clinical Key, Scopus, Web of Science, Embase, and the Cochrane Database of Systematic Reviews. See the supplementary file for detailed search strategies specific to each database.

### Studies selection and eligibility criteria

The main inclusion criteria for this systematic review, structured according to the PICOS framework, are as follows:

Population (P): Pregnant women.

Intervention (I): Pilates exercises and physical activity.

Comparison (C): No activity.

Outcomes (O): Duration of labor, active phase duration, second stage duration.

Study Design (S): Randomized Controlled Trials (RCT)/Clinical Controlled Trials (CCT).

Additionally, the studies must be; published in a peer-reviewed journal up to June 2023, and written in the English language.

Two authors (A.H. and H.Z.) initially screened the titles and abstracts of all retrieved records, and subsequently, reviewed the full texts independently. In instances of uncertainty regarding whether studies met the inclusion criteria, a third researcher (F.E.) was consulted, and decisions were made by consensus. We excluded articles not published in peer-reviewed journals, encompassing conference papers, theses, dissertations, books, book chapters, and reports from non-peer-reviewed sources, as well as those in languages other than English. Furthermore, we excluded studies that did not utilize a randomized controlled trial (RCT) or clinical controlled trial design. We included all studies published from the earliest date of consideration up to June 25, 2023, to ensure the relevance and timeliness of the evidence gathered. In cases where multiple reports originated from the same study, the most comprehensive report was selected. If the full text of an article was unavailable, the information provided in the abstract was utilized. However, if the abstract did not furnish sufficient information, the article was excluded from the study. Upon completion of the search, the EndNote program was employed to eliminate duplicates. Relevance checks were performed based on both the titles and abstracts, as well as the full texts. Additionally, the references of the included studies were reviewed to identify any potentially missing relevant papers.

### Quality assessment

RCTs were included in the evaluation using the Cochrane Risk of Bias tool (version 1). This tool comprises several domains: selection, performance, detection, attrition, reporting, and other biases. The potential for bias in these assessments can be categorized as high, low, or unclear risk of bias. The quality of all included studies was independently assessed by two investigators, AH and HZ. In cases of disagreement, resolutions were sought through consultation with colleagues FA and DR.

### Data extraction

The data were extracted into a sheet (Tables 1 and 2). The extracted data included the following items: summary characteristics such as author (s), year, sample size, study design, age, body weight, BMI, height, education, gestational age at study entry, session characteristics, and duration of labor. Overall labor duration is defined length of the first and second stages of labor. The first stage of labor is the time elapsed from the beginning of contractions to 10 cm of dilation; which includes the latent phase (the beginning of contractions up to 4 cm of dilation) and the active phase (the time elapsed from 4 to 10 cm. of dilation); the second stage is the time elapsed from full dilation (10 cm of dilation) until fetal expulsion [25].

**Table 1:** Characteristics of individual studies included in the meta-analysis

Author (s), yr, reference	Country	Sampledesign, Size	Age (yr) (mean ± SD)	Body weight (kg)	BMI (kg/m²)	Height (m)	Education (*n*, %)	Gestational age at study entry (w)	Sessions number
Ghandali 2021	Iran	RCT, Pilates group, 51	25.16 ± 4.41	-	22.71 ± 1.57	-	1. Did not finish high school, 10 (19.6%). 2. Finished high school, 22 (43.1%). 3. University, 19 (36.5%).	26.71 ± 0.78	18 sessions
		Control group, 52	23.81 ± 4.30	-	22.38 ± 1.52	-	1. Did not finish high school, 9 (17.3%). 2. Finished high school, 24 (46.2%). 3. University, 19 (37.3%).	26.67 ± 0.73	
Aktan et al. 2021	Turkey	RCT, clinical Pilates exercises, 21	27.52 ± 3.88	65.66 ± 8.09	25.05 ± 2.84	161.9 ± 5.48	1. Elementary School, 0 (0%). 2. High School, 2 (9.6%). 3. University, 19 (90.4%).	39.38 ± 1.1	16 **sessions**
		Control group, 22	25.5 ± 4.19	67.31 ± 11.86	26.01 ± 3.6	160.5 ± 6.34	1. Elementary School, 4 (18.2%). 2. High School, 15 (68.2%). 3. University, 3 (13.6%).	39.05 ± 1.36	
Rodriguez-Blanque et al. 2019	Spain	RCT, Pilates group, 60	-	75.35 ± 12.13	27.76 ± 4.03	1.646 ± 0.06	-	32.12 ± 43	51 sessions
		Control group, 60	-	79.05 ± 11.64	29.03 ± 4.45	1.651 ± 0.05	-	30.58 ± 4.75	
Barakat et al. 2018	Spain	RCT, Pilates group, 176	-	-	23.37 ± 3.73	-	Primary school 28(12.3%)، Secondry school 85(37.4%)، tertiary education 114(50.3%)	31.77 ± 4.56	85 sessions
		Control group, 149	-	-	23.69 ± 3.78	-	Primary school 71(35.1%)، Secondry school 86(42.6%)، tertiary education 45(22.3%)	31.25 ± 3.36	
Perales et al. 2016	Spain	RCT, Pilates group, 83	-	-	23.1 ± 3.2	-	Elementary 16(19.3%)، High school 40(48.2%) T university/college 27(32.5%)	31.4 ± 3.7	93 sessions
		Control group, 83	-	-	24.0 ± 4.0	-	Elementary 19(22.9%)، High school 35(42.2%) T university/college 29(34.9%)	31.8 ± 4	
Haakstad 2020	Norway	RCT, Pilates group, 43	31.2 ± 3.6	67.9 ± 11.4			College education ≥ 4 years 44.2%	17.3 ± 4.1	(60 min 2/w)
		Control group, 47	30.3 ± 4.4	67.3 ± 12.4			college education ≥ 4 years 35.8%	18.0 ± 4.3	
Mazzarino et al. 2022	Australia	RCT, Pilates group, 11	-	73.3 ± 12.9	26.1 ± 3.8	167 ± 5.7	-	39.3 ± 1	
		Control group, 10	-	63.5 ± 7.6	23.3 ± 2.8	164.9 ± 8.7	-	38 ± 0.71	
Ferreira et al. 2019	Brazil	quasi-experimental study, Intervention, 99	32.0 ± 3.6	-	23.5 ± 3.4	-	-	12w − 15w	10 sessions
		Control, 156	30.7 ± 4.2	-	24.2 ± 4.2	-	-	12w − 15w	
Price 2012	USA	RCT, Intervention, 31	30.5 ± 5	-	26.6 ± 3.1	-	-	12w -14w	96 sessions
		Control, 31	27.6 ± 7.3	-	28.7 ± 5.4	-	-	12w -14w	
Bolanthakodi et al. 2018	India	RCT, Intervention, 75	23.98 ± 3.39	54.68 ± 6.15	23.06 ± 2.33	153.89 ± 3.54	Upto 12th 56(74.66%) Graduate 19(29.33%)	30	7 sessions (30 min)
		Control, 75	23.45 ± 3.42	54.57 ± 4.94	22.92 ± 1.62	154.21 ± 3.18	Upto 12th 62(82.66%) Graduate 13(17.33%)	30 w	
Gehan 2015	Egypt	CCT, Intervention, 30	36.56	-	27.46	-	-	14 w	29 sessions
		Control, 30	36.83	-	28.27	-	-	14 w	

**Table 2:** Specifications of studies included in the meta-analysis of labor duration (sum of active phase and second stage), active phase, and second stage

	Author (s), yr, reference	Sample size Mean ± SD period of active phase (min)							Mean ± SD period of Second stage (min)					Mean ± SD Duration of labor (min)				
		Intervention group			Control Group			Value (p)	Intervention group		Control Group		Value (p)	Intervention group		Control Group		Value (p)
		N	Mean	SD	N	Mean	SD		Mean	SD	Mean	SD		Mean	SD	Mean	SD	
1	Ghandali 2021	51	110	70.94	52	164	99.81	0.004	33.49	24.51	50.36	38.59	0.52	170.42	70.94	99.81	247.54	0.004
2	Aktan 2021	21	-	-	22	-	-	-	-	-	-	-	-	439.00	238.9	555.00	299.04	0.821
3	Rodriguez-Blanque et al. 2019	60	-	-	60	-	-	-	-	-	-	-	-	389.33	216.18	561.30	199.94	< 0.001
4	Barakat et al. 2018	176	409.15	185.74	149	462.83	208.37	0.01	33.23	22.53	36.21	25.93	0.68	442.37	188.72	215.84	499.04	0.01
5	Perales et al. 2016	83	399.1	322.1	83	537.4	409.3	0.01	40.6	42.8	37.4	44.7	0.87	-	-	-	-	-
6	Haakstad 2020	21	408	330	53	588	324	0.029	49.4	35.2	49.3	27.1	0.987	457.4	330	637.3	32**4**	0.029
7	Mazzarino 2022	11	-	-	10	-	-	-	-	-	-	-	-	302	241	455	190	0.04
8	Ferreira et al. 2019	99	174	120	156	186	126	0.584	35.8	35.6	33.4	31	0.701	-	-	-	-	-
9	Price 2012	31	564	300	31	504	204	0.33	47.4	36	28.4	12.5	0.05	611	316	532	214	0.33
10	Bolanthakodi et al. 2018	75	607	143.4	75	717.6	180.6	< 0.001	57.6	27.6	62.04	25.2	0.31	675.6	157.68	790.8	188.4	< 0.007
11	Gehan 2012	30	321.6	110	30	362.4	164	0.026	-	-	-	-	-	-	-	-	-	-

### Data analysis

Statistical analysis was conducted using Review Manager (RevMan) version 5.4 and STATA version 17. A significance level was set at a *P* value of less than 0.05. Overall effects were synthesized through the mean and standard deviation of labor length. Heterogeneity among studies was assessed using the I-squared test (I²). If the I² value exceeded 50%, outlier studies were first removed to homogenize the study sample. When heterogeneity persisted, random-effects models were utilized for the analysis instead of fixed-effect models. Publication bias was assessed using Egger’s test.

### Equity, diversity, and inclusion statement

Our research team is dedicated to promoting diversity, equity, and inclusion in clinical practice, research, and training programs. Accordingly, the processes of data extraction, study selection, quality assessment, analysis, and interpretation were conducted independently to ensure results that accurately reflect the most diverse and realistic picture possible. We incorporated data from all primary research worldwide that met the inclusion criteria, without bias towards race, ethnicity, culture, socioeconomic status, or geographical regions.

## Result

### Study flow

**Fig. 1:**
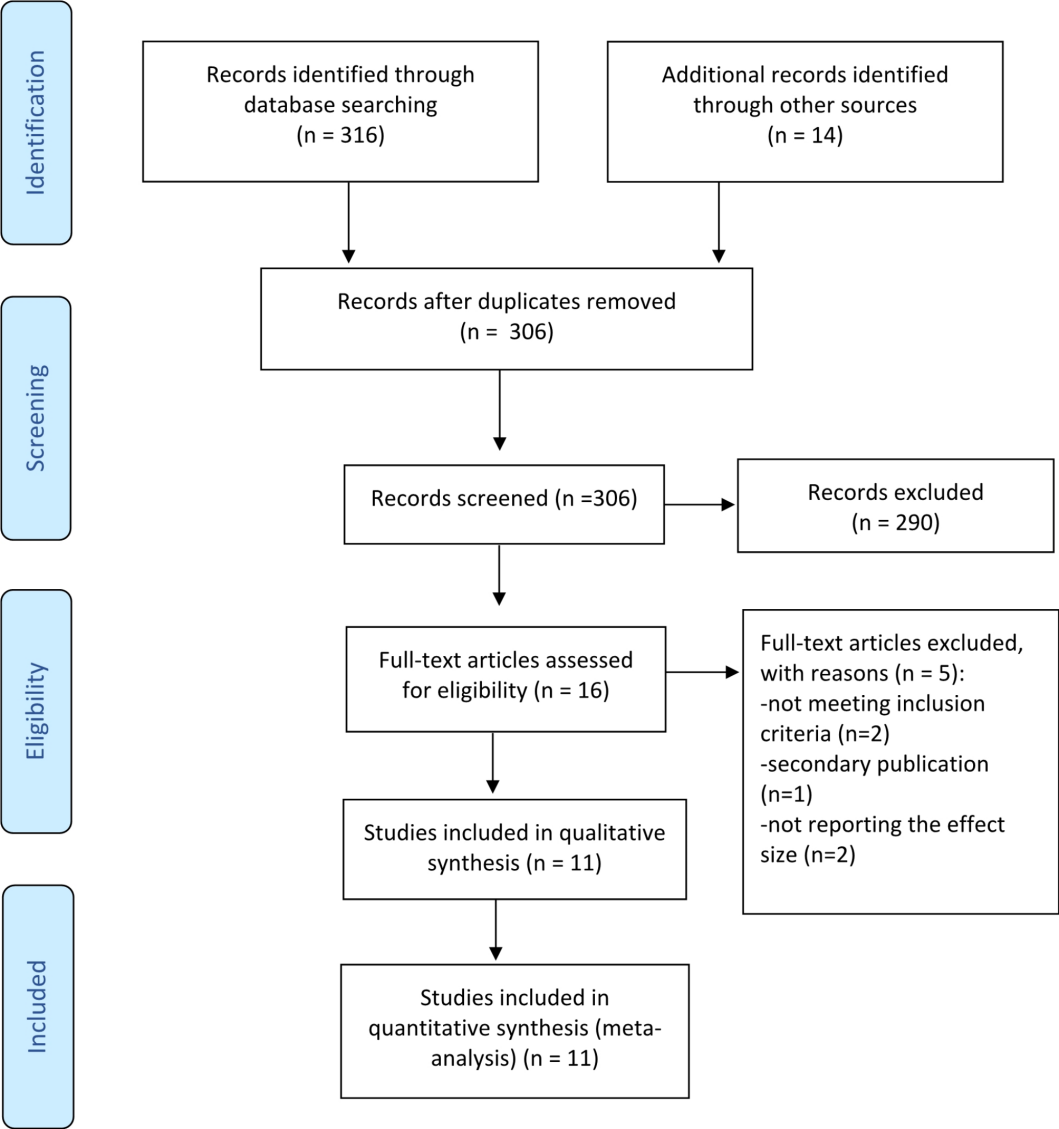
PRISMA flow diagram of the screening procedure

Figure 1 presents a chart detailing the flow of studies through this meta-analysis. In total, 330 records were screened, comprising 316 studies identified through database searches and 14 studies identified through other sources. After the removal of duplicates (24 studies), 290 studies did not meet the inclusion criteria and were subsequently excluded. The remaining 16 articles were retrieved in full for further examination. Of these, eleven studies met the inclusion criteria. The remaining five studies were excluded for the following reasons: two were cohort studies, one was a secondary publication, and two studies did not report the effect size.

### The quality assessment (risk of bias)

The authors have meticulously noted and classified the biases associated with the research methodologies into three distinct categories: low risk, high risk, and unclear risk. *High Risk*: Aktan [14], Ferreira [26], Gehan [27], Perales [28], and Mazzarino [29] were categorized as high risk due to methodological limitations, potential biases, and data reliability issues.

#### Low Risk

Barakat [30], Bolanthakodi [31] and Haakstad [32] exhibited robust methodology, adequate sample sizes, and clear results, justifying their low-risk categorization.

#### Unclear risk

**Fig. 2:**
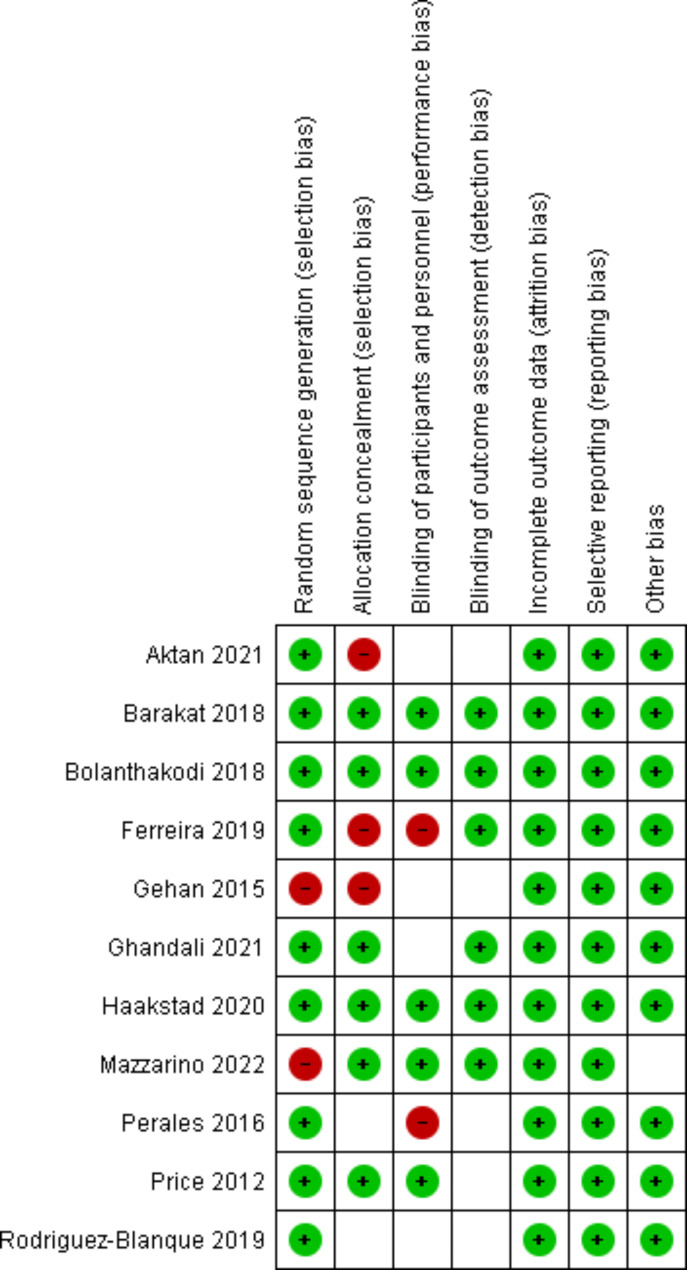
Risk of bias graph for studies

Ghandali [9], Rodriguez-Blanque [33] and Price [34] was categorized as uncertain due to ambiguities in its methodology and potential unaddressed confounding factors. This classification adheres to the criteria set forth by the Cochrane Collaboration tool. Detailed insights into this categorization, along with an exhaustive breakdown of the types and instances of bias present in the study methodologies, are comprehensively illustrated in Fig. 2.

### Study characteristics

Relevant information from the 11 selected samples is concisely summarized in Table 2. The meta-analytic sample comprised a total of 1239 participants, with 602 in the intervention group and 637 in the control group. The mean age of the studied participants ranged from 23.4 years [31] to 36.8 years [35]. Three studies were conducted in Spain [28, 30, 33], while the remaining were carried out in Turkey [14], Australia [29], Norway [32], Iran [9], Brazil [26], the USA [34], India [31], and Egypt [35]. All study designs were Randomized Control Trials, except for two studies which were Clinical Control Trials [26, 35]. The number of exercise sessions in the studies varied from 7 [31] to 96 [34]. Some studies reported only weight [32], or body mass index (BMI) [9, 26, 28, 30, 34, 35], while others included all of metrics (weight, height, and BMI) [14, 29, 31, 33]. Three studies have presented only the duration of labor [14, 29, 33], while other studies have reported the duration of the active phase, the second stage, and/ or the duration of labor separately [9, 26, 28, 30–32, 34, 35] (Tables 1 and 2). No studies reported an increased risk of adverse birth outcomes from Pilates exercises among previously inactive, healthy women.

### Quantitative data synthesis

The pooled effect size of Pilates exercises indicates a significant reduction in duration of the active phase (Mean Difference [MD] = -56.35, 95% Confidence Interval [CI] [-89.46 to -23.25], *p* < 0.001) with a sample size of 1195.

**Fig. 3:**
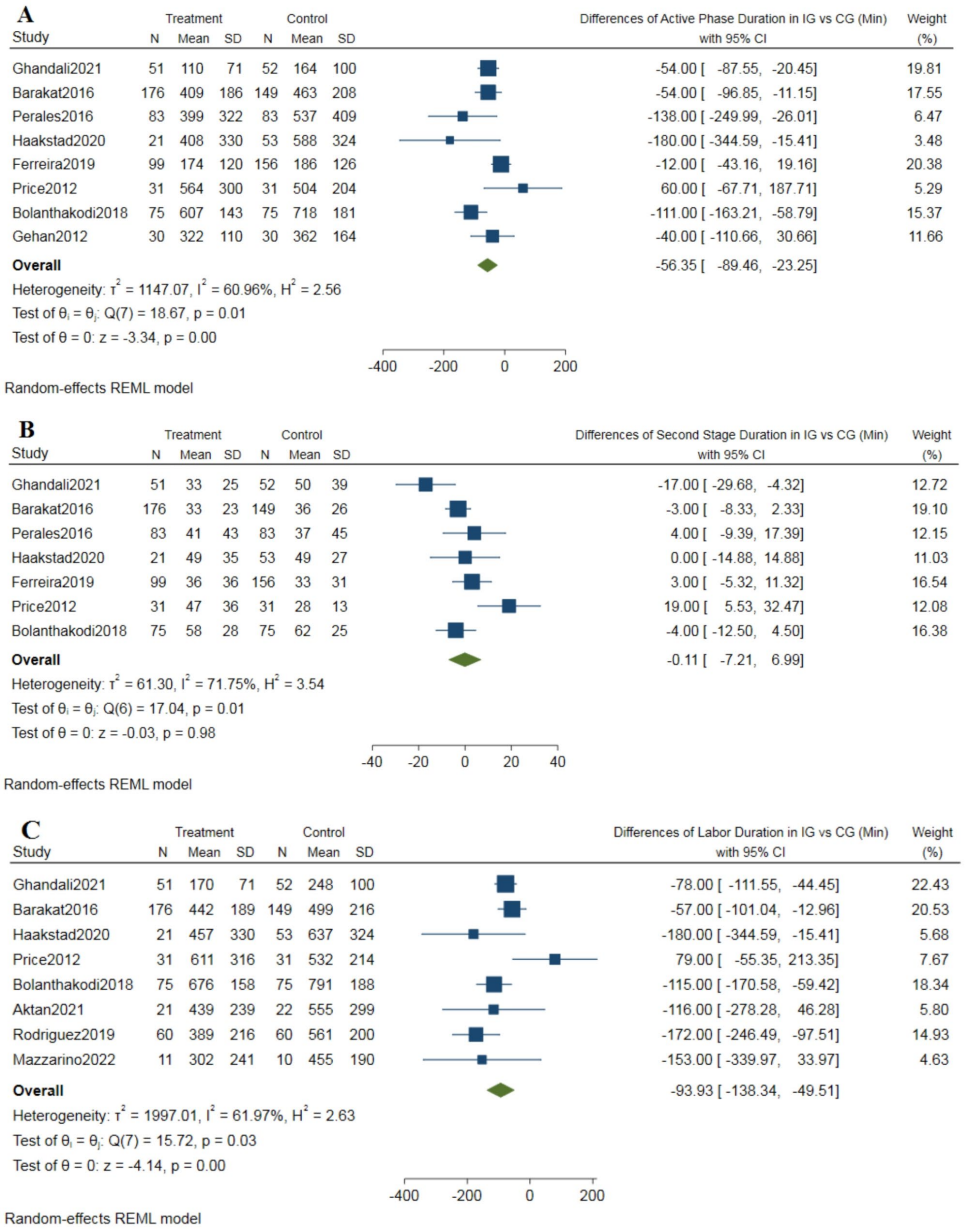
**A**; Forest plot of duration of the active phase of labor, **B**; Forest plot of duration of the second stage of labor, **C**; Forest plot of duration of labor (sum of active phase and second stage)

The mean difference in active phase of labour (− 56.35) means that Pilates reduces the duration of active phase of labor by 56.35 min in the intervention group compared to the control group. The analysed data were heterogeneous (I² = 60.96%), hence random-effects models were utilized for the analysis, but the heterogeneity could not be fully resolved (see Fig. 3A).

The Pilates exercises didn’t decrease the duration of the second stage of labor (MD = -0.11, 95% CI [-7.21 to 6.99], *P* value = 0.98), in a sample of 1135 pregnant women. The pooled estimate was homogeneous (I² = 71.75%, using a random-effect model) (refer to Fig. 3B).

Eight articles investigated the overall duration of labor (sample size = 898 pregnant women). The data indicated that the Pilates group experienced a shorter duration of labor compared to the control group (MD = -93.93, 95% CI [-138.34 to -49.51], *P* value = 0.001). The results showed heterogeneity (I² = 61.97%), and this heterogeneity could not be resolved (refer to Fig. 3C).

### Assessment of publication bias

**Fig. 4:**
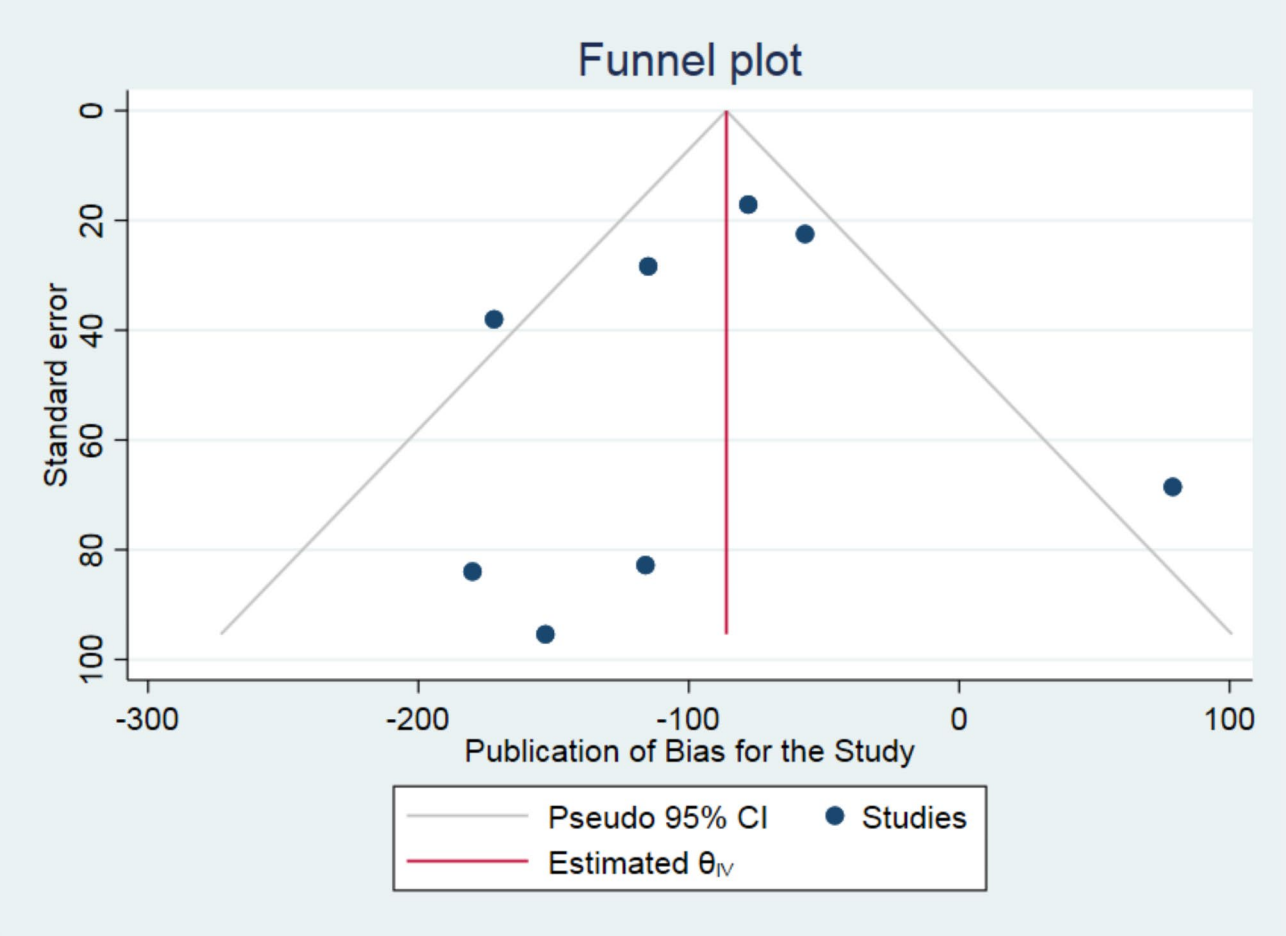
Presentation of funnel plot

We did not observe publication bias for the study in any of the variables studied. The estimated bias coefficient of labor duration was − 0.29 (Egger bias B = − 0.30 (95% CI: − 2.66–2.08) with a standard error of 0.97, giving a *p*-value of 0.77. Thus, the test provides no evidence for the presence of small-study effect. Thus, the test provides no evidence for the presence of small-study effect. Figure 4 presents the funnel plot result with the 95% confidence limit. The estimated bias coefficient of active phase duration was − 0.85 (Egger bias B = − 0.88 (95% CI: − 3.19–1.50) with a standard error of 0.96, giving a *p*-value of 0.41 and it was 1.17 (Egger bias B = 0.50 (95% CI: − 4.82–7.16) with a standard error of 2.33 (*p*-value = 0.64) for second stage of labor.

## Discussion

The present study aimed to systematically review the literature regarding the efficacy of Pilates in reducing labor length. This meta-analysis was conducted using optimal methods for secondary analysis and included 11 primary studies, all of which were clinical trials. The analytical results indicate that Pilates exercises can significantly shorten the overall duration of labor, including both the active phase and the second stage.

The meta-analysis revealed a notable difference between the Pilates exercise group and control group in the duration of the first stage of labor, despite contradictory findings in the primary studies. The disparity in results from these primary studies could be attributed to variations in sample sizes (ranging from 60 to 325) and the timing of intervention initiation (between 12 and 39 weeks’ gestation). A detailed examination of the primary studies revealed that Pilates exercises were generally more effective in studies characterized by longer durations, higher frequency, and earlier commencement of the intervention [28]. Therefore, under these specific conditions, Pilates appears to strengthen pelvic muscles, increase pelvic diameter, relax muscles, and consequently enhance the condition of the birth canal parts. This, in turn, contributes to the shortening of the active phase of labor [ 29].

The findings of this study demonstrate that Pilates doesn’t affect the duration of the second stage of labor. This conclusion confirm with some earlier studies which indicated no significant difference in second stage’s length between women who practiced Pilates and those who did not [14]. It seems that the length of the second stage of labor, which is defined from the dilatation of the cervix to the expulsion of the fetus, is more influenced by the cephalo-pelvic proportion, birth weight, the strength of the mother’s perineal tissue, and station at complete dilatation [36] as well as differences in obstetric population characteristics and variation in clinical practice [37].

In this study, Pilates was observed to reduce the overall length of labor. Supporting this finding, the outcomes of several meta-analyses have underscored the positive impact of exercise programs on facilitating the childbirth process, particularly in shortening the duration of delivery [38]. However, there exists a divergence of opinion among researchers on this matter. For instance, the findings of two separate meta-analyses suggest no significant association between the regularity, intensity, and duration of exercise and the length of childbirth [39, 40]. This discrepancy may be attributable to the type of exercise. Veisy et al., for example, focused on the influence of aerobic exercise on labor duration [39]. Contrastingly, Pilates, a non-aerobic form of exercise, is primarily recognized for its capacity to enhance physical, mental, and motor functions [15].

This form of exercise encompasses a series of low-impact routines designed to enhance strength and flexibility across the body. Regular participation in these exercises has been demonstrated to fortify the pelvic floor muscles and improve their structural functionality [41]. The enhancement of central and pelvic floor muscle strength, flexibility, and the adoption of proper breathing techniques through Pilates are known to facilitate the birthing process [9, 42]. Additionally, Pilates, initially conceptualized as a body conditioning method termed ‘Contrology’, is founded on six key principles: breath, centering, concentration, control, precision, and flow [43]. Individual investigations into each of these elements have shown that they collectively contribute to a reduction in the duration of childbirth [44, 45].

Notably, the impact of Pilates exercise on labor duration has not been previously analyzed in a systematic review or meta-analysis with a sample size of this scale. The breadth of studies and the substantial number of participants involved in this analysis afford some of the most valid and reliable conclusions that can be drawn on this subject.

### Clinical implications

Considering that prolonged labor presents a significant clinical challenge in contemporary midwifery practice, leading to various complications for both mother and child, one effective strategy to enhance labor comfort is the reduction of labor duration, specifically through methods that alleviate the duration of pain. The findings of this review indicate that practicing Pilates during pregnancy serves as a viable approach to decreasing labor length. Pilates, characterized as a discipline that bolsters physical, mental, and motor capacities, comprises a series of low-impact exercises aimed at strengthening and increasing flexibility throughout the body [15]. Analysis of primary studies reveals that Pilates can be reduced labor duration.

To date, there have been no reports of risks associated with moderate-intensity exercise for either the mother or the infant [46]. In light of this, low-risk pregnant women are encouraged to engage in progressive aerobic and resistance exercises before, during, and after childbirth. Nevertheless, it is advisable for pregnant women to undergo clinical evaluation before initiating any exercise program to ensure there are no medical contraindications to continuing with the exercise [47]. Engaging in Pilates exercises of moderate intensity during pregnancy has been shown to facilitate the delivery process.

Our findings endorse the capability of Pilates exercise to reduce the duration of labor in pregnant women. Therefore, Pilates could potentially be incorporated into childbirth preparation classes as a beneficial practice.

### Limitations

This study encountered several limitations, both at the level of the individual studies and the review itself. A notable limitation within the primary studies was the lack of reported data on the intensity of the Pilates exercises. Additionally, variations in the initiation timing and duration of exercise sessions may have influenced the results of the analysis. Another limitation at the study level was the heterogeneity of the samples. For instance, while some studies had exercises performed under professional supervision, others involved participants practicing at home without such oversight. Moreover, the participant demographics varied across studies, with many focusing on primiparous women, while others included both multiparous and primiparous participants. This variability, particularly in terms of parity, was not accounted for as a potential confounding factor, potentially contributing to the high heterogeneity observed in the meta-analyses. Consequently, the results of this study should be interpreted with caution.

At the review level, the mean effect size of the active phase period and labour duration was analysed using a random-effects model due to the heterogeneity of the studies. Furthermore, this review was limited to scientific papers published in English, representing another constraint on its scope.

## Conclusion

The evidence presented herein underscores that engaging in Pilates exercises during pregnancy is not only safe but also beneficial in optimizing the delivery process and shortening labor duration. Consistent with the guidelines of the American College of Obstetricians and Gynecologists (ACOG), this study reinforces the recommendation that, despite physiological and anatomical changes during pregnancy, women should be encouraged to maintain physical activity. Pilates, as demonstrated, can shorten length of labor. It is recommended that midwives emphasize the use of this exercise in childbirth preparation classes to reduce the duration of childbirth.

### Electronic supplementary material

Below is the link to the electronic supplementary material.

Supplementary Material 1

Supplementary Material 2

## Data Availability

Data is provided within the manuscript or supplementary information files.
