# Supplementary material for: Optimizing labor duration with pilates: evidence from a systematic review and meta-analysis of randomized controlled trials
Source: BMC Pregnancy Childbirth. 2024 Aug 31;24:573. doi: 10.1186/s12884-024-06785-5 (PMC11365214; doi:10.1186/s12884-024-06785-5)
Supplement: Supplementary file 2 — Supplementary Material 2 [file 12884_2024_6785_MOESM2_ESM.docx]

**Search strategies for included databases:**

**PubMed**

("childbirth"[Title/Abstract] OR "pregnancy"[Title/Abstract] OR "pregnant"[Title/Abstract] OR "labor"[Title/Abstract] OR "obstetric"[Title/Abstract])

AND

("Pilates"[Title/Abstract] OR "physical activity"[Title/Abstract] OR "exercise"[Title/Abstract] OR "movement techniques"[Title/Abstract])

AND

("duration of labor"[Title/Abstract] OR "length of labor"[Title/Abstract] OR "length of delivery"[Title/Abstract])

**Clinical Key**

(childbirth OR pregnancy OR pregnant OR labor OR obstetric)

AND

(Pilates OR "physical activity" OR exercise OR "movement techniques")

AND

("duration of labor" OR "length of labor" OR "length of delivery")

**Scopus Search Strategy:**

(childbirth OR pregnancy OR pregnant OR labor OR obstetric)

AND

(Pilates OR "physical activity" OR exercise OR "movement techniques")

AND

("duration of labor" OR "length of labor" OR "length of delivery")

**Web of Science Search Strategy:**

(childbirth OR pregnancy OR pregnant OR labor OR obstetric)

AND

(Pilates OR "physical activity" OR exercise OR "movement techniques")

AND

("duration of labor" OR "length of labor" OR "length of delivery")

**Embase Search Strategy:**

('childbirth':ab,ti OR 'pregnancy':ab,ti OR 'pregnant':ab,ti OR 'labor':ab,ti OR 'obstetric':ab,ti)

AND

('Pilates':ab,ti OR 'physical activity':ab,ti OR 'exercise':ab,ti OR 'movement techniques':ab,ti)

AND

('duration of labor':ab,ti OR 'length of labor':ab,ti OR 'length of delivery':ab,ti)

**Cochrane Database of Systematic Reviews Search Strategy:**

(childbirth OR pregnancy OR pregnant OR labor OR obstetric)

AND

(Pilates OR "physical activity" OR exercise OR "movement techniques")

AND

("duration of labor" OR "length of labor" OR "length of delivery")
